# Biocontrol ability and volatile organic compounds production as a putative mode of action of yeast strains isolated from organic grapes and rye grains

**DOI:** 10.1007/s10482-020-01420-7

**Published:** 2020-05-05

**Authors:** Renata Choińska, Katarzyna Piasecka-Jóźwiak, Beata Chabłowska, Justyna Dumka, Aneta Łukaszewicz

**Affiliations:** Department of Fermentation Technology, Institute of Agricultural and Food Biotechnology, 36 Rakowiecka, 02-532 Warsaw, Poland

**Keywords:** Anti-mold activity, Yeast antagonist, Biofungicide, Plant pathogens

## Abstract

The inhibiting activity of three yeast strains belonging to *Pichia kudriavzevii*, *Pichia occidentalis*, and *Meyerozyma quilliermondii*/*Meyerozyma caribbica* genera against common plant pathogens representing *Mucor* spp., *Penicillium chrysogenum*, *Penicillium expansum*, *Aspergillus flavus*, *Fusarium cereals*, *Fusarium poae*, as well as *Botrytis cinerea* genera was investigated. The yeast strains tested had a positive impact on growth inhibition of all target plant pathogens. The degree of inhibition was more than 50% and varied depending on both the yeast antagonist and the mold. Ethyl esters of medium-chain fatty acids, phenylethyl alcohol, and its acetate ester prevailed among the analyzed volatile organic compounds (VOCs) emitted by yeasts in the presence of the target plant pathogens. Due to the method used, assuming no contact between the antagonist and the pathogen, the antagonistic activity of the yeast strains studied resulted mainly from the production of biologically active VOCs. Moreover, the antagonistic activity was not only restricted to a single plant pathogen but effective towards molds of different genera, making the yeast strains studied very useful for potential application in biological control.

## Introduction

Pathogenic fungi decrease the nutritional value of food and feed. Moreover, some of them produce mycotoxins that are toxic to humans and animals. Fungal diseases and growth of pathogens are most often controlled with the use of synthetic fungicides. The main drawbacks of their use, however, include their high toxicity, ineffectiveness against certain pathogens, and problems with their registration in individual countries. The harmful effect of synthetic fungicides on the natural environment and human health has prompted the search for an eco-friendly alternative in plant disease control with particular attention to the application of microorganisms.

The use of microorganisms (yeast, yeast-like organisms, bacteria, and molds) in controlling postharvest decay of crops and fruit commodities has, actually, become one of the most frequently studied strategies for plant and harvest protection. A microbial agent having antagonistic activity against plant fungal pathogens serves as a feasible tool that allows eliminating or at least reducing synthetic fungicide usage in plant protection, by utilizing the potential of microorganisms against fungal infections/spoiling of crops and fruits during storage. Moreover, the use of microbial agents is consistent with consumers demand for fungicide/pesticide-free system of agriculture. Among microorganisms used as biological control agents (BCAs), many yeast species have proved highly effective owing to their widespread occurrence on plant materials, safety, and ability to colonize many environmental niches (Druvefors et al. 2005; Lahlali et al. 2011; Spadaro and Droby 2016). Important technological properties of yeast making them attractive and competitive in producing commercial BCAs, are rapid growth in relatively simple media/substrates with high efficiency, ability to survive in adverse environmental conditions, and being amenable to formulation with a long shelf-life (Chanchaichaovivat et al. 2007; Liu et al. 2014).

A number of yeast species have been identified as microbial antagonists and their inhibiting effect on mold growth and/or the production of mycotoxins, preventing their accumulation or reducing their level in the environment, has been described (Cao et al. 2013; Coehlo et al. 2007; Fiori et al. 2014). The frequently reported yeast antagonists include mainly strains belonging to the *Pichia* genus, e.g. *Pichia caribbica*, *Pichia guiliermondii*, *Pichia membranifaciens*, *Wickerhamomyces anomalus* (formerly *Pichia anomala*), and *Meyerozyma gulliermondii* (formerly *Pichia guilliermondii*) (Druvefors et al. 2005; Farkas et al. 2012; Lima et al. 2013; Parafati et al. 2015). Many studies have shown also the biocontrol ability of other yeast species, such as *Rhodotorula glutinis*, *Rhodotorula mucilaginosa* (Castoria et al. 2005; Li et al. 2011; Robiglio et al. 2011), *Metschnikowia pulcherrima* (Saravanakumar et al. 2008), *Saccharomyces cerevisiae* (Nally et al. 2015), *Candida saitoana*, *Candida intermedia* (Huang et al. 2011), *Hanseniaspora uvarum* (Liu et al. 2010), *Hanseniaspora opuniatae* (Ruiz-Moyano et al. 2016), and yeast-like *Aureobasidium pullulans* (black yeast) (Di Francesco et al. 2015).

The antagonistic action of yeast against pathogens can be due to different mechanisms, including competition for nutrients, colonization, production of antifungal compounds, induced systemic resistance, and mycoparasitism process (Morath et al. 2012). Saravanakumar et al. (2008) revealed that iron depletion by yeast under low iron conditions might reduce the growth of some postharvest pathogens. The following modes of action have been reported in the studies on the mechanisms of biocontrol activity of *Saccharomyces* and non-*Saccharomyces* yeasts against fungi: competition for nutrients and space (including siderophore production), reduction in spore germination, decreased germ tube length, and inhibition of mycelial growth by volatile and diffusible metabolites (Nally et al. 2015). It has also been found that some killer toxins secreted by yeast are active not only against sensitive yeasts but also against molds (Guyard et al. 2002; Lima et al. 2013; Olstorpe and Passoth 2011). Moreover, volatile organic compounds produced by microbial antagonists are effective against many postharvest fungal pathogens (Freimoser et al. 2019). Several studies have dealt with the fungistatic activity of volatile metabolites to identify and characterize their mode of action (Pretscher et al. 2018; Wan et al. 2008; Wang et al. 2013). Volatiles are produced during diverse metabolic processes and can be classified into various chemical groups, like alcohols, esters, aldehydes, ketones, and lactones. Based on the previously reported results, it has been demonstrated that volatile metabolites are involved in the competition mechanism and play an important role in the systemic resistance against predators, parasites, and diseases (Siddiquee et al. 2012). Volatile inhibitory compounds are superior over the non-volatile ones because they can diffuse to greater distances in a structurally heterogeneous environment composed of solids, liquid, and gases (Fialho et al. 2010). The majority of studies on the use of microbial VOCs in inhibiting the growth of pathogens have been carried out under laboratory conditions. Moreover, they were mainly focused on the identification of biocontrol agents against postharvest pathogens during storage. The number of reports addressing the application of microbial VOCs under open field conditions is, however, limited (Irtwange 2006; Cortes-Barco et al. 2010; Song and Ryu 2013). The use of microbial VOCs in the open field depends on several factors, such as soil properties, microbial community, and plants exudates (Fincheira and Quiroz 2018). Another relevant issue is the manner of microbial VOCs application allowing for their efficient delivery to crop plants. The lastly reported studies focused on the reproduction of lab results in the open filed tests have shown limited protection of microbial VOCs against plant pathogens (Song and Ryu 2013). The effective exploitation of microbial VOCs still remains a challenge, and more research needs to be done in this respect (Kanchiswamy et al. 2015).

The main objectives of this study were the selection of new yeast strains, isolated from fruit (organic grape) and rye grain, and the investigation of their antagonistic mode of action against molds, in terms of the production of volatile organic compounds. It also included the examination of the activity of the biocontrol agents selected against molds isolated from various plant environments to evaluate the broad spectrum of their activity, beneficial for further application in plant disease control.

## Materials and methods

### Microorganisms and culture conditions

Yeast strains used in this study were previously isolated from fruits and crops from organic farming and have been included to the Culture Collection of Industrial Microorganisms, Institute of Agricultural and Food Biotechnology (IAFB, Warsaw, Poland).

The preliminary screening for the anti-mold activity of yeast was done against molds of the *Penicillium*, *Aspergillus*, and *Fusarium* genera isolated from diseased grass silage and crops acc. to Piasecka-Józwiak and Chablowska (2017). Based on the results of the initial screening, yeast strains showing the best activity were selected for the study.

In the next step, the inhibition ability of the selected yeast strains was tested against new molds, isolated from infected grains representing the *Mucor* spp., *Penicillium chrysogenum*, *Penicillium expansum*, *Aspergillus flavus*, *Fusarium cereals*, *Fusarium poae*, and *Botrytis cinerea* genera, isolated from infested grape berries.

The three selected yeast isolates with the highest BCA (biocontrol ability) were identified, based on the D1/D2 domain sequence of the 26S rRNA gene using the primer pair NL1 and NL4 (O’Donnell 1993), as: *Pichia kudriavzevii* KKP 3005 (Kurtzman et al. 2008) isolated from rye grains (GeneBank accession number MK881743), *Pichia occidentalis* KKP 3004 isolated from organic grape (GeneBank accession number MK850405), and *Meyerozyma quilliermondii* (formerly *P. guilliermondii*)/*Meyerozyma caribbica* KKP 3003 isolated from organic grape (GeneBank accession number MK920148). The identification of molds was based on the partial sequencing of internally transcribed spacer regions and 5.8S rDNA, using ITS1 and ITS4 primers (White et al. 1990). This pair of primers has also been used in re-analyses and confirmation of yeast strains identification.

All microorganisms were stored in an atmosphere of liquid nitrogen (at − 195.8 °C). The yeast and mold stock cultures were maintained at 4 °C on Petri dishes containing Yeast Extract Peptone Dextrose Agar (YEPD yeast extract, 10 g; peptone, 10 g; dextrose, 20 g; agar, 20 g per liter of distilled water) and YEPD with 100 mM citrate phosphate buffer.

The antagonistic ability of yeast was investigated in vitro on the YGC medium (yeast extract 5 g, glucose 20 g, chloramphenicol 0.1 g per liter of distilled water, agar 14.9 g) (Merck).

### Antagonistic activity on agar plates

The tests were conducted by simultaneous co-incubation of yeast and molds. Yeast suspensions were prepared by inoculating 100 mL of a YEPD broth with 1 mL of yeast from a culture stored in YEPD at 4 °C and agitated on a rotary shaker at 25 °C for 24 h. Yeast cells were pelleted by centrifugation, re-suspended in sterile distilled water, counted in the Thoma chamber, and used to inoculate the YGC agar. The yeast suspensions were adjusted to 10^6^ spores/mL in liquid YGC agar on Petri dishes (9 cm diam). Each plate contained 10 mL of the medium.

Inoculums of mold were prepared from 7- to 9-day cultures grown in YGC at 25 °C. Spores from the cultures were collected in sterile water with 0.05% (v/v) Tween 80. Spore concentration was counted in the Thoma chamber and adjusted to 10^6^ spores/mL. 10 μL of spore suspensions were spotted in the center of Petri dishes previously inoculated with the yeast strain tested, and incubated at 25 °C. For certain molds (*B. cinerea*, *Fusarium graminearum* M26, *F. poae* M28, *Mucor* sp.), if spores were not obtained after 9-day cultivation, mycelial discs (3 mm square plug) of mycelium were placed in the center of the agar plates inoculated with the yeast. A control dish inoculated only with mold was also prepared and served as the control.

After 10 days of incubation at 25 °C, the fungal diameter was measured and growth reduction (n) was calculated compared to mold growth of the control as follows: %I = [(C − T)/C] × 100, where %I represented the inhibition of colony diameter growth, C was diameter growth measurement in control, and T was the diameter growth of the pathogen in the presence of yeast strains.

The tests were performed in three independent experiments, each in duplicate.

### Antifungal volatile compounds assay

A double Petri dish assay was used for the analysis of volatiles. To this end, a yeast suspension from a 24-h yeast culture in the YGC medium was prepared in saline water in a concentration of 10^8^ spores/mL. The yeast suspensions were adjusted to 10^6^ spores/mL in YGC agar on Petri dishes (9 cm diam), containing 15 mL of the medium. The mold spores were harvested from the culture on the solid medium and resuspended in peptone water. Spore concentration was counted in the Thoma chamber and adjusted to 10^5^ spores/mL. 10 μL of spore suspensions were spotted in the center of Petri dishes (9 cm diam) containing 15 mL of the YGC medium. The Petri dishes were left for 90 min at room temperature. Afterwards, the mold-containing plate was placed over the yeast-containing plate. Both plates were immediately sealed with parafilm and incubated at 25 °C for 5 days.

Comparable analyses were conducted on the Petri dishes containing only molds, yeasts as well as an uninoculated medium. The compounds identified in the samples containing the uninoculated medium and molds were subsequently subtracted from the analysis to show only compounds produced by the yeasts studied.

#### Extraction and identification of volatile potentially anti-mold compounds

Volatiles produced at the headspace above the mold cultures were collected using the solid-phase microextraction (SPME) technique (Strobel et al. 2001; Zhang and Pawliszyn 1993). After 5 days of incubation, an SPME syringe containing Carboxen-PDMS on a stable fiber (75 µm) (Supelco) was gently inserted through a small hole drilled in the parafilm and exposed to the vapor phase for 45 min at room temperature. Then, the syringe was pulled out and inserted into the injection port of a gas chromatograph (Varian model 3800, Walnut Creek, USA) connected to an ion-trap mass spectrometer (Saturn 2000 model, Varian) for 30 s. A DB-5MS column (30 m × 0.25 mm × 0.25 µm, J&W Scientific, Folsom, CA, USA) was used for the separation of the volatiles. The oven temperature was programmed as follows: 50 °C for 1.8 min, followed by a linear increase of 5 °C/min to a maximum of 220 °C and held for 1 min. Helium was used as the carrier gas at the rate of 1 mL/min. Injector temperature was 170 °C. The manifold, GC/MS interface, and ion trap temperatures were set at 50 °C, 250 °C, and 180 °C, respectively. Mass spectra were obtained using electron impact ionization (70 eV). Scanning was performed from m/z 35 to 200 in the electronic impact mode.

Volatile compounds were tentatively identified by comparison of their mass spectra with those available in the NIST 98 MS database.

### Data analysis

The statistical analyses were conducted using the Statistica version 8 software. The significance of differences in mean values of colony’s diameters were established using the one-way analysis of variance (Anova) and Tukey’s post hoc tests. All analyses were performed at α = 0.05.

## Results and discussion

### Evaluation of in vitro antagonism

The antagonistic ability of the three yeast strains, i.e. *P. kudriavzevii* KKP 3005, *P. occidentalis*/*Issatchenkia orientalis* KKP 3004, and *M. quilliermondii*/*Meyerozyma caribbica* KKP 3003, against molds isolated from infected grains and grapes was investigated in vitro on the YGC medium. The results obtained showed that all three yeast strains studied were highly active against the target molds representing *Fusarium* sp., *Mucor* sp., *B. cinerea*, *Penicillium* sp., and *Aspergillus fumigatus* species (Table 1). The inhibition of mold growth, expressed as a reduction of colony growth, was higher than 50% compared to controls, while in the case of *P. kudriavzevii* KKP 3005 it was well above 80%. The degree of mold inhibition varied depending on the pathogen and yeast antagonist. Among the studied yeast, *M. quilliermondii*/*M. caribbica* KKP 3003 was the least effective against *Penicilium chrysogenum* and *Fusarium sp*., whereas *P. occidentalis* KKP 3004 and *P. kudriavzevii*/*I. orientalis* KKP 3005 inhibited the growth of these molds at a similar level. In the case of *Mucor* sp. and *B. cinerea*, growth reduction reached about 85%, and no significant differences were observed among the yeast strains studied.

**Table 1:** Antagonistic activity of yeast against mold (expressed as growth reduction %, mean ± standard error)

Molds	*Meyerozyma guilliermondii*/*M. caribbica*	*Pichia occidentalis*	*Pichia kudriavzevii*/*Issatchenkia orientalis*
*Penicillium chrysogenum* M5	86.5 ± 1.96 a	87.7 ± 3.35 a	92.9 ± 4.43 b
*Penicillium chrysogenum* M24	85.8 ± 2.66 a	91.2 ± 1.75 b	91.1 ± 2.13 b
*Penicillium chrysogenum* M11	99.5 ± 1.13 b	100.0 ± 0.00 b	86.0 ± 3.40 a
*Penicillium chrysogenum* M14	50.2 ± 4.73 a	71.4 ± 1.28 b	95.2 ± 3.17 c
*Penicillium crustosum* M9	98.6 ± 1.54 c	53.2 ± 1.81 a	83.7 ± 5.35 b
*Penicillium commune*/*expansum* M6	77.0 ± 1.86 b	52.1 ± 2.28 a	81.5 ± 3.27 c
*Fusarium cereals* M3	66.7 ± 3.05 a	81.9 ± 2.21 b	88.6 ± 1.99 c
*Fusarium graminearum* M25	88.3 ± 1.33 a	91.4 ± 1.26 b	91.6 ± 2.22 b
*Fusarium graminearum* M26	76.5 ± 2.13 b	71.8 ± 2.00 a	92.7 ± 1.32 c
*Fusarium poae* M28	79.8 ± 1.50 a	89.5 ± 1.50 c	85.4 ± 1.43 b
*Aspergillus fumigatus*	82.7 ± 3.79 a	97.8 ± 1.88 c	87.8 ± 3.23 b
*Mucor* sp.	85.9 ± 1.30 a	84.8 ± 1.76 a	85.8 ± 2.36 a
*Botrytis cinerea*	83.4 ± 1.20 a	82.3 ± 1.43 a	82.4 ± 2.60 a

Different letters indicate significant differences among the mean values according to the least significant difference test (*P* ≤ 0.05)

### Volatile organic compounds (VOCs)

The study on the inhibitory effect of the volatile compounds emitted by the yeast strains studied, as a putative mode of their action against target molds, was performed using the double Petri dish assay, assuming no contact between the antagonist and the mold. The volatile compounds collected in the gas phase were tentatively identified using SPME coupled with the GC–MS technique, which is commonly used for the analysis of volatile profiles of microorganisms (Jeleń 2003; Stoppacher et al. 2010). After 5 days of incubation at 25 °C, the growth of molds tested was distinctly inhibited (well above 60%), except for *B. cinerea*, where the percentage of its growth reduction was the lowest (~ 11%). In the other cases, the growth reduction was similar to that observed on the YGC medium, which proves explicitly the antagonistic activity of VOCs released by the yeasts studied (Table 2).

**Table 2:** Antagonistic activity of VOCs emitted by yeast strains studied against target molds (expressed as growth reduction %, mean ± standard error)

Molds	*Meyerozyma guilliermondii*/*M. caribbica*	*Pichia occidentalis*	*Pichia kudriavzevii*/*Issatchenkia orientalis*
*Penicillium chrysogenum* M24	73.3 ± 1.31 a	90.5 ± 1.45 b	91.3 ± 2.10 b
*Fusarium poae* M28	75.4 ± 1.04 a	85.3 ± 1.76 b	84.8 ± 1.32 b
*Aspergillus fumigatus*	84.2 ± 1.98 a	95.2 ± 1.81 c	90.1 ± 2.54 b
*Mucor* sp.	64.7 ± 1.54 a	72.6 ± 1.12 b	73.5 ± 1.85 b
*Botrytis cinerea*	11.1 ± 2.20 a	11.8 ± 1.06 a	11.2 ± 1.25 a

Different letters indicate significant differences among the mean values according to the least significant difference test (*P* ≤ 0.05)

The identified volatile compounds were grouped into six families, such as esters, alcohols, terpenes, ketones, aldehydes, and aromatic hydrocarbons. Table 3 presents possible compounds identified in the gas phase above the yeast cultured alone, while Tables 4, 5 and 6 summarize possible compounds identified in the headspace above molds culture after 5-day exposure to yeast gases. Ester family, including mainly ethyl esters of medium-chain fatty acids, dominated over the other groups, especially in the case of *P. occidentalis* KKP 3004 (16 compounds) and *P. kudriavzevii*/*I. orientalis* KKP 3005 (10 compounds). The emitted volatiles were strain-specific however, some of them were the same. An aromatic alcohol, phenylethyl alcohol, and its acetate ester have been identified in the case of all three yeast strains tested. The production of phenylethyl alcohol, phenylethyl acetate, and ethyl esters of the medium-chain fatty acids has been reported in other yeast species as well, e.g.: *Candida tropicalis*, *S. cerevisiae*, and *P. kudriavzevii* (Fiahlo et al. 2011; Koumba Kone et al. 2016). These compounds act mainly as aroma compounds, however phenylethyl alcohol and phenylethyl acetate have also been recognized as effective antifungal volatile compounds (Arrarte et al. 2017; Liu et al. 2014; Masoud and Kaltoft 2006; Meshram et al. 2017; Ting et al. 2010). Di Francesco et al. (2015) described the antagonistic activity of phenylethyl alcohol and other alcohols emitted by *A. pullulans* against five fruit postharvest pathogens. Results of their study showed that the identified VOCs, at a given concentration, were highly effective in suppressing the growth of pathogens. Phenylethyl alcohol was one of the identified antifungal metabolites of endophytic fungus *Muscor albus*, an effective biocontrol agent in controlling post-harvest diseases of fruit crops (Strobel et al. 2001). Wan et al. (2008) detected phenylethyl alcohol among other volatile compounds emitted by filamentous bacteria *Streptomyces platensis* F-1, a potential biofumigant to control plant fungal diseases. In turn, phenylethyl acetate produced by *P. anomala*, *Pichia kluyveri*, and *Hanseinaspora uvarum* has been shown to strongly inhibit the growth of *Aspergillus ochraceus* (Masoud et al. 2005).

**Table 3:** Volatile organic compounds (VOC’s) identified in the headspace of *Pichia occidentalis*, *Pichia kudriavzevii*, and *Meyerozyma guilliermondii* after 5 days of incubation at 25 °C

Yeast	Volatile organic compounds					
	Aldehydes	Alcohols	Esters	Ketones	Terpenes	Others
*Pichia occidentalis* KKP 3004	Benzaldehyde .alpha.Campholenal 2-phenyl-1-propanal	Benzyl alcohol Phenylethyl alcohol 2-hexyl-1-decanol 2-hexyl-1-octanol	Diamyl carbonate Ethyl hexenoate Isoamyl butanoate Ethyl heptanoate Ethyl octanoate Phenethyl acetate Ethyl 9-decenoate Ethyl decanoate Isoamyl octanoate Ethyl dodecanoate Isoamyl decanoate Ethyl tetradecanoate Isobutyl benzoate	Acetophenone 3-hexanone, 2,2-dimethyl Ethanone,1-(2,4-dimethylphenyl) Cyclohexanone,4-(1,1-dimethylethyl)	.alpha.Limonene d-Verbenone *trans*-Carveol .beta.Farnesene	Pyrazine, 3-ethyl-2,5-dimethyl Azulene Furan,3-phenyl Benzene,1,3,-bis(1,1-dimethylethyl) Nonadecane
*Pichia kudriavzevii*/*Issatchenkia orientalis* KKP 3005	2-nonenal	Phenylethyl alcohol 1-dodecanol,3,7,11 –trimethyl 2-hexyl-1-decanol 2-nonanol,5-ethyl 2-hexyl-1-octanol	Isoamyl butanoate Ethyl butanoate Amyl propionate Ethyl hexanoate Ethyl octanoate Phenethyl acetate Ethyl decanoate Isoamyl octanoate Ethyl dodecanoate Isoamyl decanoate	4-octen-3-one 2-Tetradecanone		7,11-dimethyloctadecane
*Meyerozyma guilliermondii*/*Meyerozyma caribbica* KKP 3003	Tetradecanal	2-decen-1-ol Phenylethyl alcohol Isogeraniol 9-octadecenol	Isoamyl butanoate Phenethyl acetate Citronellyl formate Ethyl dodecanoate Ethyl nonanonate Methyl dihydrojasmonate	Acetophenone 2-Nonanone	*o*-Cymene alpha-Limonene .alpha.Bisabolol .beta.Farnesene .alpha.Himachalene	Benzene,1,3,-bis(1,1-dimethylethyl

Analyses were performed with SPME and GC–MS, using 75 µm Carboxen™-PDMS SPME fiber

**Table 4:** Volatile organic compounds (VOCs) identified in the headspace above mold culture after 5 days exposure to *Pichia occidentalis* KKP 3004 gases at 25 °C. Analyses were performed with SPME and GC–MS, using 75 µm Carboxen™-PDMS SPME fiber

Molds	Volatile organic compounds					
	Aldehydes	Alcohols	Esters	Ketones	Terpenes	Others
*Penicillium chrysogenum* *Botrytis cinerea* *Fusarium poae* *Aspergillus fumigatus* *Mucor* sp.	Benzaldehyde Benzenacetaldehyde .alpha.Campholenal 3,5-di *tert*-butyl-4-hydroxybenzaldehyde	Benzyl alcohol Phenylethyl alcohol 2-hexyl-1-decanol 3-Penten-1-ol,2,2,4-trimethyl 2-hexyl-1-octanol 2-hexyl-1-decanol	Diamyl carbonate Ethyl isobutanoate Isoamyl butanoate Ethyl heptanoate Ethyl octanoate Phenethyl acetate Ethyl nonanoate Ethyl 9-decenoate Ethyl decanoate Isoamyl octanoate Ethyl dodecanoate Isoamyl decanoate Ethyl tetradecanoate Isobutyl benzoate Ethyl hexadecenoate	2*H*-inden-2-one,1,3-dihydro Acetophenone 2-nonanone 3,6-dimethyl-4-octanone 3-hexanone, 2,2-dimethyl 2-decanone Cyclohexanone,4-(1,1-dimethylethyl	.alpha.Limonene .beta.Farnesene .alpha.Curcumene .beta.Bisabolene Neoclovene Germacrene D	Ethanone,1,2-diphenyl-, oxime 1-heptene,4-methyl Furan,3-phenyl 2,2,4,-trimethyl-5(2,2-dimethylpropyl)-3(2*H*)-furanone Benzene,1,3,-bis(1,1-dimethylethyl) Furan,3-ethyl-2,5-dihydro Pyrazine,2,3,5,-tris(1,1-dimethylethyl) Pyrazine, 3-ethyl-2,5-dimethyl Nonadecane 7,11-dimethyloctadecane 1-hexene,4-ethyl 1,4-Naphthalenedione, 2-(methyl)-3-(2-methyl-2-propenyl)

**Table 5:** Volatile organic compounds (VOCs) identified in the headspace above mold culture after 5 days exposure to *Pichia kudriavzevii* KKP 3005 gases at 25 °C

Molds	Volatile organic compounds					
	Aldehydes	Alcohols	Esters	Ketones	Terpenes	Others
*Penicillium chrysogenum* *Botrytis cinerea* *Fusarium poae* *Aspergillus fumigatus* *Mucor* sp.	Benzenacetaldehyde 1-heptanal, 3,5,5-triethyl Tetradecanal	Phenylethyl alcohol 2-hexyl-1-decanol 2-hexyl-1-octanol	Diamyl carbonate Ethyl isobutanoate Isoamyl butanoate Ethyl hexanoate Ethyl octanoate Phenethyl acetate Ethyl decanoate Isoamyl octanoate Ethyl dodecanoate Ethyl 2-heptenoate Isoamyl decanoate Ethyl dodecanoate Ethyl tetradecanoate	1,2-propanedione, 1-phenyl Acetophenone 2-Nonanone 2-decanone	Cedrene	Tetradecane 7,11-dimethyloctadecane 1-heptene,4-methyl Benzene,1,3,-bis(1,1-dimethylethyl) Pyrazine, 3-ethyl-2,5-dimethyl Nonadecane

Analyses were performed with SPME and GC–MS, using 75 µm Carboxen™-PDMS SPME fiber

**Table 6:** Volatile organic compounds (VOCs) identified in the headspace above mold culture after 5 days exposure to *Meyerozyma guilliermondii* KKP 3003 gases at 25 °C

Molds	Volatile organic compounds					
	Aldehydes	Alcohols	Esters	Ketones	Terpenes	Others
*Penicillium chrysogenum* *Botrytis cinerea* *Fusarium poae* *Aspergillus fumigatus* *Mucor* sp.	3-hydroxy-4-methylbenzaldehyde 1-heptanal, 3,5,5-triethyl 2,2-dimethyl propanal	Phenylethyl alcohol 2-hexyl-1-decanol 2-butyl-1-octanol Isogeraniol 1-dode-canol,3,7,11-trimethyl	Diamyl carbonate Ethyl isobutanoate Isoamyl butanoate Isoamyl acetate Ethyl octanoate Phenethyl acetate Ethyl nonanoate Ethyl pentadecanoate	Acetophenone 2-Nonanone	Neoclovene *o*-Cymene Himachalene .beta.Farnesene	Tetradecane 1-heptene,4-methyl Benzene,1,3,-bis(1,1-dimethylethyl) Pyrazine, 3-ethyl-2,5-dimethyl

Analyses were performed with SPME and GC-MS, using 75 µm Carboxen™-PDMS SPME fiber

The identified volatiles emitted by *P. occidentalis* KKP 3004 and *M. quilliermondii*/*M. caribbica* KKP 3003, included also terpenes (including mono-and sesquiterpene). Some authors have indicated the biological potential of sesquiterpenes in the VOCs-mediated antagonistic interactions (Li et al. 2018). Limonene, a beta-bisabolene found in this study, was also detected in the volatile profiles of *Trichoderma* isolates exhibiting an antagonistic effect against the causal agent of barley diseases (Moya et al. 2018). Hence, the same types of volatiles could be produced by different species. However, both the production and the antimicrobial activity of VOCs varied among species and depended on several factors, including culture conditions, media type, and antagonist cell concentration.

This study showed, for the first time ever, that the VOCs produced by *P. kudriavzevii* KKP 3005, *P. occidentalis*/*I. orientalis* KKP 3004, and *M. quilliermondii*/*M. caribbica* KKP 3003, were effective against common pathogens belonging to different genera. The results obtained confirm the previous findings reported by several authors who investigated the antagonistic activity of VOCs and, therefore, they are another evidence emphasizing the essential role of VOCs in the inhibitory effect of yeast. Additionally, our study demonstrated a high efficiency of the yeasts used towards a broad spectrum of molds. It should be emphasized that, due to its limited nature, the identification technique employed allowed only the general identification of the components of the headspace above the mold cultures. Investigations performed by other authors revealed that the detection of generated volatiles and their percentage could be influenced by the analytic methodology applied and current physiological state of the antagonist (Di Francesco et al. 2015; Siddiquee et al. 2012). The widely described VOCs-mediated antagonistic action of microbials has been mostly assayed based on the observed degree of inhibition of mold growth after exposure to antagonists headspace. Thus, the production of VOCs is species-specific and, as it has already been mentioned, the antimicrobial action of volatiles should be treated as a synergistic effect with other suggested inhibitory mechanisms against the pathogen.

## Conclusion

The results of this study prove that the volatile compounds released by the three yeast strains studied acted antagonistically against molds isolated from infected crop/plants, effectively inhibiting their growth. The demonstrated activity of the selected yeast strains against plant molds well predicts their potential use in fungicidal preparations. Thus, *P. kudriavzevii* KKP 3005, *P. occidentalis*/*I. orientalis* KKP 3004, and *M. quilliermondii*/*M. caribbica* KKP 3003 can serve as promising alternatives to synthetic fungicides in the control of fungal diseases of plants. Further studies are, however, needed to develop a method for their preservation in commercial preparations.
